# Omega Class Glutathione S-Transferase: Antioxidant Enzyme in Pathogenesis of Neurodegenerative Diseases

**DOI:** 10.1155/2017/5049532

**Published:** 2017-12-24

**Authors:** Youngjo Kim, Sun Joo Cha, Hyun-Jun Choi, Kiyoung Kim

**Affiliations:** ^1^Soonchunhyang Institute of Medi-bio Science, Soonchunhyang University, Cheonan 31151, Republic of Korea; ^2^Department of Medical Biotechnology, Soonchunhyang University, Asan 31538, Republic of Korea

## Abstract

The omega class glutathione S-transferases (GSTOs) are multifunctional enzymes involved in cellular defense and have distinct structural and functional characteristics, which differ from those of other GSTs. Previous studies provided evidence for the neuroprotective effects of GSTOs. However, the molecular mechanisms underpinning the neuroprotective functions of GSTOs have not been fully elucidated. Recently, our genetic and molecular studies using the *Drosophila* system have suggested that GstO1 has a protective function against H_2_O_2_-induced neurotoxicity by regulating the MAPK signaling pathway, and GstO2 is required for the activation of mitochondrial ATP synthase in the *Drosophila* neurodegenerative disease model. The comprehensive understanding of various neuroprotection mechanisms of *Drosophila* GstOs from our studies provides valuable insight into the neuroprotective functions of GstOs *in vivo*. In this review, we briefly introduce recent studies and summarize the novel biological functions and mechanisms underpinning neuroprotective effects of GstOs in *Drosophila*.

## 1. Introduction

Glutathione S-transferases (GSTs) are a superfamily of multifunctional isoenzymes involved in the cellular detoxification of several endogenous and exogenous compounds. GSTs catalyze the nucleophilic attack of glutathione (GSH) on the electrophilic centers of substrates, including insecticides, toxic compounds, metabolites, and organic hydroperoxides. GSTs play a crucial role against carcinogens, therapeutic drugs, and various types of cellular oxidative damage [1]. GSTs also regulate the biosynthesis and intracellular transport of hormones [1]. Based on their sequence similarity and substrate specificities, GSTs are subdivided into at least ten subfamilies: alpha, delta, epsilon, kappa, mu, pi, sigma, theta, zeta, and omega [2].

The omega class GST (GSTO) is the most recently defined GST class and a relatively ancient cytosolic enzyme [3, 4]. GSTOs appear to be widespread in nature and have been identified in bacteria, insects, yeast, mammals, and plants [2, 3, 5–7]. GSTO shares low sequence similarity with other GST classes but exhibits the GST fold. GSTO has interesting characteristics compared with those of other GST types. The active sites of GSTOs have a cysteine residue at the N-terminus that can bind to GSH, whereas other GST classes have tyrosine or serine residues in their active sites [8]. GSTOs have distinct enzymatic properties and thiol transferase and dehydroascorbate (DHA) reductase activities, which are similar to reactions catalyzed by thioredoxin and glutaredoxin [3]. There is increasing evidence that GSTOs are also involved in the detoxification of several exogenous stressors. Silkmoth GSTO was induced in the fat body after exposure to several environmental stressors, including bacteria and ultraviolet-B (UV-B) [9]. GSTO3 from the human pathogenic filarial worm *Onchocerca volvulus* (*Ov*GSTO3) demonstrates stress-resistant effects [10]. Overexpressing GSTO-1 in *Caenorhabditis elegans* exhibits increased resistance during oxidative damage [11]. In addition, GSTOs were shown to scavenge free radicals by regulating DHA reduction and catalyzing the reduction of inorganic arsenic, monomethylarsonate (MMA), and dimethylarsonate (DMA) [12–14]. *In vitro* studies have shown that human GSTO1 participates in modulation of the ryanodine receptor, which is a Ca^2+^ release channel. In addition, these studies also showed that human GSTO1 is involved in modulation of the signaling pathway during c-Jun N-terminal kinase- (JNK-) mediated apoptosis and in the activation of interleukin-1*β*, an important mediator of inflammatory response [15–17]. Human GSTO1-1 is a novel regulator of lipopolysaccharide- (LPS-) induced inflammatory responses in macrophages and is required for LPS-mediated signaling in macrophages [18, 19].

Recently, an important role for human GSTO1-1 in glutathionylation of the target proteins has been described [17, 20]. *β*-Actin has been reported to be deglutathionylation by human GSTO1-1 [20]. GSTO1-1 decreased global protein glutathionylation level in macrophages [18]. These findings indicate a critical role for GSTO1 in redox homeostasis through affecting glutathionylation/deglutathionylation of the target proteins. Furthermore, genetic polymorphisms in the human *GSTO1* and *GSTO2* genes may be associated with the risk of bladder, urothelial, breast, and ovarian cancer [21, 22]. Thus, GSTOs play important roles in decreasing oxidative stress produced by various stressors and cellular processes. Recent studies related to neurodegenerative disorders have implicated polymorphic variants of GSTOs in the age at onset and progression of neurodegenerative diseases such as Alzheimer's disease (AD) and Parkinson's disease (PD) [23–25]. GSTO2 expression levels are decreased in the brains of AD patients. Although GSTO has a protective function against neuronal damage, the molecular mechanisms and physiological functions of GSTOs are still not clear and should be researched further. In this review, we mainly focus on recent studies that have investigated the neuroprotective functions of GSTOs in the *Drosophila* model system.

## 2. Omega Class Glutathione S-Transferases in *Drosophila*

On the basis of the genome sequences and comparative analyses, the *Drosophila* GST genes can be divided into six subfamilies: delta, epsilon, sigma, omega, zeta, and theta. *Drosophila* harbors 36 GST genes that encode 41 proteins [2, 26]. Four different *GSTO* genes in *Drosophila* are located on chromosome 3L. *GSTO* genes form a cluster spanning approximately 6 kb [2]. This is evidence of internal duplication within the cluster, evolutionally. This duplication gave rise to differentially expressed GSTO isoforms and generated diverse members with differing functionality. These four *GSTO* genes had been named previously as follows: *sepia*, *GstO1*, *GstO2*, and *GstO3* [2]. The sequence identities/similarities are high, at 43–65%/66–82%, based on the amino acid sequence alignment of the different isoforms of GSTO in *Drosophila* [27]. All isoforms of GSTO have N-terminal extensions and cysteine residues in the active site rather than tyrosine or serine residues, which are found in the active sites of other classes of GSTs. Furthermore, all isoforms of GSTOs have high thiol transferase and DHA reductase activities, characteristic of GSTOs, and low activity toward 1-chloro-2,4-dinitrobenzene (CDNB), a general GST substrate [27]. In addition, the tissue distributions of GSTOs were determined by reverse transcription polymerase chain reaction (RT-PCR) and Western blot analysis [27–29]. They have a different tissue distribution in *Drosophila*. Sepia was found only in the eye. GstO1 and GstO2A were highly expressed in the head and abdomen of adult flies. However, GstO2B and GstO3 were expressed at approximately the same level in all tissues. Therefore, these studies suggest that *Drosophila* GSTOs might possess tissue-specific physiological functions.

## 3. Neuroprotective Functions of GstOs in *Drosophila*

### 3.1. GstO1 Has a Protective Function against Neuronal Toxicity

Oxidative stress poses a major threat to organisms living in an aerobic environment and plays a critical role in several neurological disease processes [30]. Oxidative stress is widely implicated in neuronal cell death. Hydrogen peroxide (H_2_O_2_) has been implicated in triggering apoptotic death in several cell types [31, 32]. It may also induce the production of reactive oxygen species (ROS) in neuronal cells. In a recent study, our group determined that GstO1 is highly expressed in the head, and *GstO1* mutant flies are sensitive to ROS, produced under H_2_O_2_ exposure. Interestingly, H_2_O_2_-induced lethality and apoptotic cell death of neurons in *GstO1* mutant flies were suppressed by neuron-specific expression of GstO1. These results suggest that GstO1 has a physiological function in neurons, and GstO1 neuronal expression is sufficient to suppress H_2_O_2_-induced neurotoxicity in *GstO1* mutant flies.

Phosphorylation cascades leading to the activation of MAPK, including ERK, JNK, and p38, are among the major cellular signaling pathways known to influence cell survival under ROS damage [33–35]. Several reports have shown that accumulation of ROS in response to H_2_O_2_ exposure results in the activation of several stress kinases, involving the ASK1, ERK, and JNK pathways [33–35]. Our studies of H_2_O_2_-induced neurotoxicity in *GstO1* mutant flies reveal that increased ERK phosphorylation in *GstO1* mutant flies treated with H_2_O_2_ was rescued by the expression of GstO1 [28]. The mechanism for the suppression of H_2_O_2_-mediated neurotoxicity appears to be mediated through the suppression of ERK pathway activation. Thus, these findings strongly demonstrate that GstO1 has a critical, protective role against H_2_O_2_-mediated neurotoxicity by regulating the MAPK pathway.

### 3.2. GstO2 Regulates Complex V Activity in Neurodegenerative Disease

Previous studies have reported that single-nucleotide polymorphisms in human *GSTO* genes are associated with the age at onset for AD, PD, and stroke [36, 37]. The *GSTO1 D140* allele is associated with a decreased risk of familial PD [23]. In addition, a possible relationship between GSTOs loci and the age at onset of amyotrophic lateral sclerosis (ALS) has been reported [38]. These various studies provide evidence that genetic variation of human *GSTOs* can influence the age at onset of several different neurodegenerative diseases. These studies also suggest that GSTOs may contribute to the pathogenesis of each neurological disorder and have a protective role in neuronal cells during the development of neurological diseases. However, many studies have failed to demonstrate the molecular function of GSTOs *in vivo*. A speculative mechanism involving the pathogenesis of neurodegenerative disease was recently proposed. Our subsequent study showed that GstO2A is a novel genetic regulator of the *Drosophila parkin* mutant, which is the popular *Drosophila* model of PD [39]. Furthermore, we showed compelling evidence that GstO2A catalyzes the glutathionylation of the ATP synthase *β* subunit, which is a catalytic component of the mitochondrial ATP synthase complex (complex V). The glutathionylation of the ATP synthase *β* subunit induced by GstO2A expression in *parkin* mutants is important for the rescue of ATP synthase activity in these mutants [39]. Moreover, human GSTO1 has the capacity to glutathionylate or deglutathionylate target proteins [20]. Although the role GstO2A plays in the glutathionylation of target proteins is not clear, these findings strongly suggest that enhancing the activity of GstO2A in neuronal cells could alleviate neurodegeneration in the *Drosophila* model of PD.

### 3.3. GstO2 Regulates Ascorbic Acid Recycling

Ascorbic acid (AsA), the reduced form of vitamin C, is an essential cofactor in various enzymatic reactions. AsA is an important antioxidant with multiple cellular functions and plays a role in detoxification against endogenous and exogenous stressors. Interestingly, the brain exhibits one of the highest AsA concentrations in the body. AsA concentrations of 1~2 mM have been detected throughout the brain while intracellular concentrations in neuronal cells are much higher, reaching up to 10 mM [14]. This evidence suggests a critical role of AsA in the brain or neuronal cells [40]. Imbalance of AsA homeostasis has also been demonstrated in neurodegenerative diseases such as AD, PD, and ALS.

In most cells, ascorbic acid is regenerated from the oxidized form of ascorbic acid, DHA [41, 42]. This recycling pathway of DHA to AsA is known to be mediated by specific reductases, such as GSH- or NADPH-dependent DHA reductases [43–45]. AsA is synthesized in the liver of several mammals. However, humans and other primates do not express the specific enzyme for AsA biosynthesis and are unable to synthesize AsA [46]. Therefore, humans require a supply of AsA from food. In this regard, understanding the mechanism for AsA recycling is important for maintaining cellular AsA homeostasis. Previous reports have shown that the DHA reductase activity of human GSTO2 is approximately 70–100-fold higher than that of human GSTO1 using an *in vitro* enzyme assay [12]. The notable feature of human GSTO2 is very high DHA reductase activity, which suggests that human GSTO2 may have a protective role against oxidative stress by recycling AsA [12]. In *Drosophila*, GstO2B has the highest GSH-dependent DHA reductase activity among the GstOs [27]. In addition, we showed that GSH-dependent DHA reductase activity is decreased in *GstO2* mutant flies. Furthermore, the AsA redox state, determined by the AsA/DHA ratio, was also dramatically decreased in *GstO2* mutants [39]. These studies suggest that GstO2B may be critical in the maintenance of AsA concentrations in cells and plays a protective role against oxidative stress by regulating the AsA recycling pathway in *Drosophila*.

## 4. Structural Difference and Diverse Function of Omega GSTs in *Drosophila*

**Figure 1:**
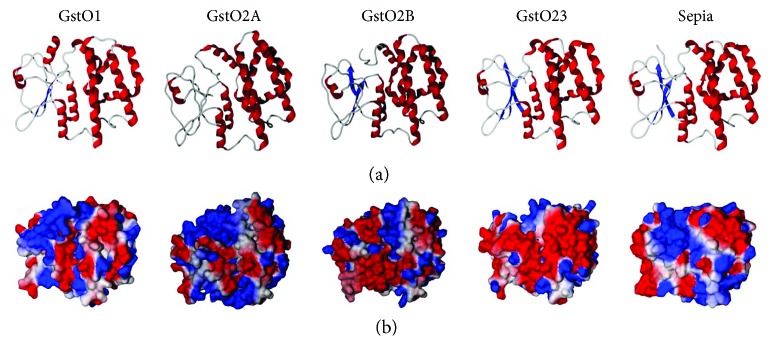
Comparison of the three-dimensional (3D) structures and surface electrostatic potential distributions of *Drosophila* GstOs. The 3D structure predictions of GstOs were generated by I-TASSER server for protein structure prediction, which is based on a threading alignment algorithm (a). Overall 3D ribbon structures of GstOs are shown (b). Negative and positive charges are represented in red and blue, respectively. The figures were generated by the Molegro Molecular Viewer.

Binding of the GSH to GSTs is highly conserved in the N-terminal domain. Substrate-binding sites (H-site) in the C-terminal domain of GSTs are variable with different features, hydrophobicities, shapes, and electrostatic potential distributions [47]. Many research groups have shown that residues in the substrate-binding sites of omega class GST homologues are well conserved across species [48–50]. Although most residues in the G-site and H-site of GstOs in *Drosophila* are highly conserved [27], the GstOs have different physiological functions *in vivo* [39, 51]. Therefore, we constructed three-dimensional models of *Drosophila* GstOs using I-TASSER server and analyzed the surface electrostatic potential distributions (Figure 1). The electrostatic potential distributions of GstO1, GstO2A, and sepia are similar, with only some differences. In contrast, the electrostatic potential distributions of GstO2B and GstO3 differ markedly from those of the other *Drosophila* GstO electrostatic potential distributions. These features are likely to be determinants of interactions between *Drosophila* GstOs and substrates that are still to be discovered. We showed that GstOs have different electrostatic potential distributions and substrate-binding site shapes from each other, by homology modeling analysis. Because these GstO isoforms differ only in the portion of the C-terminal domain that binds the hydrophobic substrate, this region may influence substrate preference. These data may explain the functional differences between each GstO isoforms. However, the differences in the functions and catalytic mechanisms of GstOs have not been fully elucidated. Identification of the differences in the structure and electrostatic potential of the GstO substrate-binding sites helped us understand the catalytic role of GstOs in reaction with different substrates and their ability to perform different functions *in vivo*.

## 5. Putative Functions of Other GSTOs in *Drosophila*

Although the *in vivo* function of *Drosophila* GstO3 is not yet elucidated, there is some evidence that it may also be involved in antioxidant processes. The *GstO3* transcript of *Drosophila* is expressed at approximately the same level in all tissues. Interestingly, the expression level of *GstO3* transcript increased in response to various stressors such as heat shock, heavy metal stress, and exposure to rotenone [52–54]. However, little is known with regard to the exact mechanism responsible for increased GstO3 expression. Thus, GstO3 may have a wide range of antioxidant activities. Further studies are required to understand the physiological function and molecular mechanism by which GstO3 protects cells from various oxidative stressors.

## 6. Conclusions and Perspective

GSTs are ubiquitously expressed enzymes belonging to the GSH-mediated antioxidant. Sequence alignment analyses revealed that GSTs, including GSTOs, exist in a wide range of organisms. The broad distribution of several GSTs among all living organisms may reflect its important and diverse physiological functions. Particularly, the role exerted by GSTOs in neuronal cells appears to be relevant. Although various studies suggest that oxidative stress plays an important role in the pathogenesis of several human diseases, including neurodegenerative diseases, the exact mechanism of GSTOs in neuroprotective effects against several oxidative stressors that provide a pharmacological basis for the relationship between GstOs and the development of neurodegenerative diseases has not been elucidated. As discussed in this review, our research on *Drosophila* GstOs has shown that GstOs are involved in protective effects against various neurotoxic conditions. For instance, GstO1 has a protective function against H_2_O_2_-induced neurotoxicity by regulating the MAPK signaling pathway. In addition, GstO2 is required for the activation of mitochondrial ATP synthase in the *Drosophila* model of PD. This finding suggests that enhancing the activity of GstO2 in neuronal cells could alleviate neurodegeneration in the *Drosophila* model of PD. Furthermore, GstO2 has a DHA reductase activity and is required for the recycling of AsA. Thus, GstO2 may play a critical role in the maintenance of AsA concentrations in neuronal cells and plays a protective role against oxidative stress by regulating the AsA recycling pathway in neuronal cells. A comprehensive understanding of various neuroprotection mechanisms of *Drosophila* GstOs and a further investigation of their pharmacological and antitoxicological properties will enhance our understanding of their role in neuronal cells and the pathogenesis of neurodegenerative diseases.

Previously, our studies provided several evidences for novel diverse roles of GstOs, based on genetic and molecular studies using the *Drosophila* model system. The C-terminal domain, including the H-site of *Drosophila* GstOs, is less similar than the N-terminal domain, which contains a cysteine residue in the G-site of GstOs. Thus, the structural differences in the C-terminal domain of GstOs may be responsible for the differences in the functions of various *Drosophila* GstOs and may influence substrate preference. Further studies are required to identify the *in vivo* substrates of GSTOs, which are related to neuroprotection, in order to better understand the functional diversity of GSTOs. Because various biological processes are conserved in *Drosophila* and mammals, we expect that the elucidation of diverse *in vivo* functions of *Drosophila* GstOs will have broad biological implications in understanding neuroprotection mechanisms.

## References

[1] Hayes J. D., Flanagan J. U., Jowsey I. R. (2005). Glutathione transferases. *Annual Review of Pharmacology and Toxicology*.

[2] Saisawang C., Wongsantichon J., Ketterman A. J. (2012). A preliminary characterization of the cytosolic glutathione transferase proteome from *Drosophila melanogaster*. *Biochemical Journal*.

[3] Board P. G., Coggan M., Chelvanayagam G. (2000). Identification, characterization, and crystal structure of the omega class glutathione transferases. *The Journal of Biological Chemistry*.

[4] Board P. G. (2011). The omega-class glutathione transferases: structure, function, and genetics. *Drug Metabolism Reviews*.

[5] Dixon D. P., Davis B. G., Edwards R. (2002). Functional divergence in the glutathione transferase superfamily in plants. Identification of two classes with putative functions in redox homeostasis in *Arabidopsis thaliana*. *The Journal of Biological Chemistry*.

[6] Garcera A., Barreto L., Piedrafita L., Tamarit J., Herrero E. (2006). *Saccharomyces cerevisiae* cells have three omega class glutathione S-transferases acting as 1-Cys thiol transferases. *Biochemical Journal*.

[7] Xun L., Belchik S. M., Xun R. (2010). S-Glutathionyl-(chloro)hydroquinone reductases: a novel class of glutathione transferases. *The Biochemical Journal*.

[8] Board P. G., Menon D. (2013). Glutathione transferases, regulators of cellular metabolism and physiology. *Biochimica et Biophysica Acta (BBA) - General Subjects*.

[9] Yamamoto K., Teshiba S., Shigeoka Y. (2011). Characterization of an omega-class glutathione S-transferase in the stress response of the silkmoth. *Insect Molecular Biology*.

[10] Liebau E., Hoppner J., Muhlmeister M. (2008). The secretory omega-class glutathione transferase *Ov*GST3 from the human pathogenic parasite *Onchocerca volvulus*. *The FEBS Journals*.

[11] Burmeister C., Luersen K., Heinick A. (2008). Oxidative stress in *Caenorhabditis elegans*: protective effects of the omega class glutathione transferase (*GSTO-1*). *The FASEB Journal*.

[12] Schmuck E. M., Board P. G., Whitbread A. K. (2005). Characterization of the monomethylarsonate reductase and dehydroascorbate reductase activities of omega class glutathione transferase variants: implications for arsenic metabolism and the age-at-onset of Alzheimer’s and Parkinson’s diseases. *Pharmacogenetics and Genomics*.

[13] Zakharyan R. A., Sampayo-Reyes A., Healy S. M. (2001). Human monomethylarsonic acid (MMA(V)) reductase is a member of the glutathione-S-transferase superfamily. *Chemical Research in Toxicology*.

[14] Rice M. E. (2000). Ascorbate regulation and its neuroprotective role in the brain. *Trends in Neurosciences*.

[15] Laliberte R. E., Perregaux D. G., Hoth L. R. (2003). Glutathione s-transferase omega 1-1 is a target of cytokine release inhibitory drugs and may be responsible for their effect on interleukin-1β posttranslational processing. *The Journal of Biological Chemistry*.

[16] Dulhunty A., Gage P., Curtis S., Chelvanayagam G., Board P. (2001). The glutathione transferase structural family includes a nuclear chloride channel and a ryanodine receptor calcium release channel modulator. *The Journal of Biological Chemistry*.

[17] Paul S., Jakhar R., Bhardwaj M., Kang S. C. (2015). Glutathione-S-transferase omega 1 (GSTO1-1) acts as mediator of signaling pathways involved in aflatoxin B1-induced apoptosis-autophagy crosstalk in macrophages. *Free Radical Biology & Medicine*.

[18] Menon D., Coll R., O’Neill L. A. J., Board P. G. (2014). Glutathione transferase omega 1 is required for the lipopolysaccharide-stimulated induction of NADPH oxidase 1 and the production of reactive oxygen species in macrophages. *Free Radical Biology & Medicine*.

[19] Menon D., Coll R., O'Neill L. A. J., Board P. G. (2015). GSTO1-1 modulates metabolism in macrophages activated through the LPS and TLR4 pathway. *Journal of Cell Science*.

[20] Menon D., Board P. G. (2013). A role for glutathione transferase omega 1 (GSTO1-1) in the glutathionylation cycle. *The Journal of Biological Chemistry*.

[21] Marahatta S. B., Punyarit P., Bhudisawasdi V., Paupairoj A., Wongkham S., Petmitr S. (2006). Polymorphism of glutathione S-transferase omega gene and risk of cancer. *Cancer Letters*.

[22] Pongstaporn W., Rochanawutanon M., Wilailak S., Linasamita V., Weerakiat S., Petmitr S. (2006). Genetic alterations in chromosome 10q24.3 and glutathione S-transferase omega 2 gene polymorphism in ovarian cancer. *Journal of Experimental & Clinical Cancer Research*.

[23] Allen M., Zou F., Chai H. (2012). Glutathione S-transferase omega genes in Alzheimer and Parkinson disease risk, age-at-diagnosis and brain gene expression: an association study with mechanistic implications. *Molecular Neurodegeneration*.

[24] Capurso C., Panza F., Seripa D. (2010). Polymorphisms in glutathione *S*-transferase omega-1 gene and increased risk of sporadic Alzheimer disease. *Rejuvenation Research*.

[25] Board P. G., Menon D. (2016). Structure, function and disease relevance of omega-class glutathione transferases. *Archives of Toxicology*.

[26] Enayati A. A., Ranson H., Hemingway J. (2005). Insect glutathione transferases and insecticide resistance. *Insect Molecular Biology*.

[27] Kim J., Suh H., Kim S., Kim K., Ahn C., Yim J. (2006). Identification and characteristics of the structural gene for the *Drosophila* eye colour mutant *sepia*, encoding PDA synthase, a member of the omega class glutathione S-transferases. *Biochemical Journal*.

[28] Lee S. Y., Lim I. A., Kang G. U. (2015). Protective effect of *Drosophila* glutathione transferase omega 1 against hydrogen peroxide-induced neuronal toxicity. *Gene*.

[29] Kim K., Yim J. (2014). Structural modelling and molecular characterization of omega-class glutathione S-transferase 2 from *Drosophila melanogaster*. *Insect Molecular Biology*.

[30] Gamaley I. A., Klyubin I. V. (1999). Roles of reactive oxygen species: signaling and regulation of cellular functions. *International Review of Cytology*.

[31] Desagher S., Glowinski J., Premont J. (1996). Astrocytes protect neurons from hydrogen peroxide toxicity. *The Journal of Neuroscience*.

[32] Desagher S., Glowinski J., Premont J. (1997). Pyruvate protects neurons against hydrogen peroxide-induced toxicity. *The Journal of Neuroscience*.

[33] Cobb M. H. (1999). MAP kinase pathways. *Progress in Biophysics and Molecular Biology*.

[34] Adler V., Yin Z., Tew K. D., Ronai Z. (1999). Role of redox potential and reactive oxygen species in stress signaling. *Oncogene*.

[35] Guyton K. Z., Liu Y., Gorospe M., Xu Q., Holbrook N. J. (1996). Activation of mitogen-activated protein kinase by H_2_O_2_. Role in cell survival following oxidant injury. *The Journal of Biological Chemistry*.

[36] Kolsch H., Linnebank M., Lutjohann D. (2004). Polymorphisms in glutathione *S*-transferase omega-1 and AD, vascular dementia, and stroke. *Neurology*.

[37] Li Y. J., Oliveira S. A., Xu P. (2003). Glutathione S-transferase omega-1 modifies age-at-onset of Alzheimer disease and Parkinson disease. *Human Molecular Genetics*.

[38] van de Giessen E., Fogh I., Gopinath S. (2008). Association study on glutathione S-transferase omega 1 and 2 and familial ALS. *Amyotrophic Lateral Sclerosis*.

[39] Kim K., Kim S. H., Kim J., Kim H., Yim J. (2012). Glutathione s-transferase omega 1 activity is sufficient to suppress neurodegeneration in a *Drosophila* model of Parkinson disease. *The Journal of Biological Chemistry*.

[40] Spector R., Lorenzo A. V. (1973). Ascorbic acid homeostasis in the central nervous system. *The American Journal of Physiology*.

[41] May J. M. (2002). Recycling of vitamin C by mammalian thioredoxin reductase. *Methods in Enzymology*.

[42] Wilson J. X. (2002). The physiological role of dehydroascorbic acid. *FEBS Letters*.

[43] Xu D. P., Washburn M. P., Sun G. P., Wells W. W. (1996). Purification and characterization of a glutathione dependent dehydroascorbate reductase from human erythrocytes. *Biochemical and Biophysical Research Communications*.

[44] Del Bello B., Maellaro E., Sugherini L., Santucci A., Comporti M., Casini A. F. (1994). Purification of NADPH-dependent dehydroascorbate reductase from rat liver and its identification with 3α-hydroxysteroid dehydrogenase. *Biochemical Journal*.

[45] May J. M., Mendiratta S., Hill K. E., Burk R. F. (1997). Reduction of dehydroascorbate to ascorbate by the selenoenzyme thioredoxin reductase. *The Journal of Biological Chemistry*.

[46] Kiuchi K., Nishikimi M., Yagi K. (1982). Purification and characterization of L-gulonolactone oxidase from chicken kidney microsomes. *Biochemistry*.

[47] Wu B., Dong D. (2012). Human cytosolic glutathione transferases: structure, function, and drug discovery. *Trends in Pharmacological Sciences*.

[48] Brock J., Board P. G., Oakley A. J. (2013). Structural insights into omega-class glutathione transferases: a snapshot of enzyme reduction and identification of a non-catalytic ligandin site. *PLoS One*.

[49] Zhang Y., Yan H., Lu W., Li Y., Guo X., Xu B. (2013). A novel omega-class glutathione *S*-transferase gene in *Apis cerana cerana*: molecular characterisation of *GSTO2* and its protective effects in oxidative stress. *Cell Stress and Chaperones*.

[50] Chen B. Y., Ma X. X., Guo P. C. (2011). Structure-guided activity restoration of the silkworm glutathione transferase omega GSTO3-3. *Journal of Molecular Biology*.

[51] Kim K., Yim J. (2013). Glutathione S-transferase omega suppresses the defective phenotypes caused by *PINK1* loss-of-function in *Drosophila*. *Biochemical and Biophysical Research Communications*.

[52] Yepiskoposyan H., Egli D., Fergestad T. (2006). Transcriptome response to heavy metal stress in *Drosophila* reveals a new zinc transporter that confers resistance to zinc. *Nucleic Acids Research*.

[53] Sorensen J. G., Nielsen M. M., Kruhoffer M., Justesen J., Loeschcke V. (2005). Full genome gene expression analysis of the heat stress response in *Drosophila melanogaster*. *Cell Stress & Chaperones*.

[54] Mod E. C., Roy S., Ernst J. (2010). Identification of functional elements and regulatory circuits by *Drosophila* modENCODE. *Science*.

